# Neuroregenerative implications of dimethylglyoxal (diacetyl): A reactive metabolite in diabetes and hyperglycemic stroke

**DOI:** 10.4103/NRR.NRR-D-25-00279

**Published:** 2026-02-05

**Authors:** Julica Inderhees, Riccardo Costalunga, Markus Schwaninger

**Affiliations:** Institute of Experimental and Clinical Pharmacology and Toxicology, University of Lübeck, Lübeck, Germany

**Figure 1:**
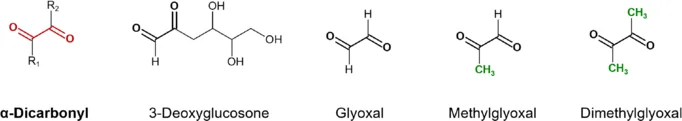
Chemical structure of α-dicarbonyls.

Dicarbonyls are biologically active compounds characterized by the presence of two adjacent carbonyl groups within the same molecule (**Figure 1**). This unique structure determines their high reactivity, presenting the basis of their biological activity. Dicarbonyls can form glycation adducts with proteins, lipids, and nucleic acids, which is why they have gained attention in diabetes research in recent years. Non-enzymatic glycation reactions occur in various tissues, leading to the formation of advanced glycation end products (AGEs) and modifications that can alter the function of the affected biomolecules. In individuals with diabetes, elevated blood glucose levels lead to increased dicarbonyl production, contributing to complications associated with the disease. The most prominent and frequently investigated representatives of the dicarbonyl group are 3-deoxyglucosone, glyoxal, and methylglyoxal (**Figure 1**). These compounds and their respective AGEs have been associated, among others, with peripheral neuropathy, nephropathy, and retinopathy (Singh et al., 2001). AGEs are known to promote inflammatory processes and oxidative stress by binding to the receptor for AGEs (RAGE), which exacerbates metabolic dysfunction in diabetes.

Next to peripheral complications, a significant concern for patients with diabetes is vascular dementia, a type of cognitive impairment caused by vascular damage in the brain (Cheng et al., 2012). However, it is important to note that brain endothelial cells, a crucial part of the blood-brain barrier, do not exclusively rely on glucose as an energy source, but also utilize lactate and pyruvate (Rhein et al., 2024). In diabetes, large portions of 3-deoxyglucosone, glyoxal, and methylglyoxal are produced directly from excess glucose or as by-products of glycolysis, making the utilization of lactate or pyruvate appear to be a safer way to meet energetic demands. Nevertheless, brain endothelial cells and neurons, which rely on lactate as well (Pellerin et al., 1998), are the cell populations most affected by neurological damage in diabetes. This inconsistency could at least partially be resolved by the discovery of a new reactive glucose metabolite – dimethylglyoxal (**Figure 1**).

The structure of dimethylglyoxal (diacetyl) has been known for a long time. It occurs naturally in butter and other natural products, contributing to their characteristic flavor. Therefore, it is frequently used as a flavoring substance, e.g. in popcorn. Interestingly, it has been described as harmful and pro-inflammatory, causing bronchiolitis obliterans in exposed factory workers (Kreiss et al., 2002). In the context of diabetes, so far it has only been measured in the respiratory air after glucose exposure; however, authors attributed the dimethylglyoxal production to bacteria in the mouth (Ghimenti et al., 2013). Bacteria and yeast are indeed known to produce dimethylglyoxal as a byproduct of branch-chain amino acid production via acetolactate synthase. Recently, it has been shown that dimethylglyoxal is an endogenous metabolite that can be detected in blood and tissue, especially after short- or long-term glucose exposure (Rhein et al., 2024). Strikingly, higher levels of the dicarbonyl dimethylglyoxal were observed in plasma and brain tissue of diabetic mice, detected and unambiguously identified by liquid-chromatography coupled to tandem mass spectrometry (LC-MS/MS). Additionally, dimethylglyoxal levels were also increased in the serum of patients with type 1 or type 2 diabetes, underlining the clinical relevance of this dicarbonyl that has been newly described as a mammalian metabolite.

**Figure 2:**
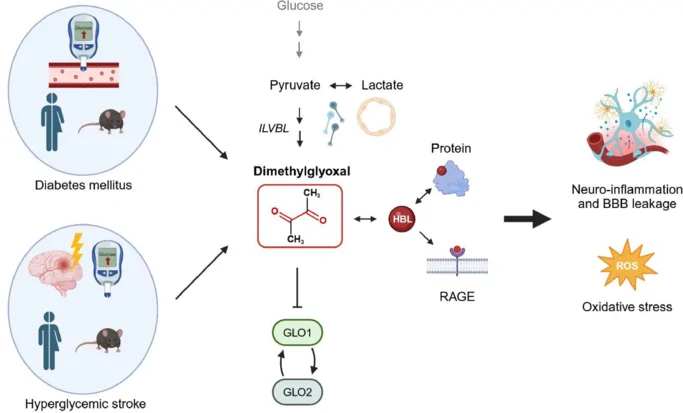
Role of dimethylglyoxal in diabetes and hyperglycemic stroke. Created with BioRender.com. BBB: Blood–brain barrier; GLO1: glyoxalase 1; GLO2: glyoxalase 2; HBL: Nε-3-hydroxy-2-butanonelysine; ILVBL: bacterial acetolactate synthase-like; RAGE: receptor for advanced glycation end products; ROS: reactive oxygen species.

Dimethylglyoxal is particularly interesting for diabetes research as it is considered even more reactive than structurally similar dicarbonyls like glyoxal or methylglyoxal due to the lower hydration rate (Hooper, 1967). While other dicarbonyls can be degraded by the glyoxalase system based on their aldehyde structure, this enzymatic pathway does not work for dimethylglyoxal, a ketone (**Figure 1**). Moreover, dimethylglyoxal is not only resistant to glyoxalase I but may also act as inhibitor against this important catabolic enzyme and thereby aggravate dicarbonyl stress (**Figure 2**; Lupidi et al., 2001). Most intriguingly, dimethylglyoxal production does not necessarily depend on glucose, but is formed from pyruvate (**Figure 2**; Rhein et al., 2024). Therefore, it is not surprising that dimethylglyoxal can be synthesized by brain endothelial cells *in vitro*. Endogenous dimethylglyoxal production could explain why this specific cell type is not protected from dicarbonyl stress *in vivo*.

The newly discovered pathway of dimethylglyoxal formation from pyruvate changes the view on reactive glucose metabolites. Cells that rely on lactate or pyruvate are not protected from dicarbonyl stress but might be even more vulnerable due to the high reactivity of dimethylglyoxal. Indeed, dimethylglyoxal exposure has been shown to promote neuro-inflammation and oxidative stress *in vitro* and *in vivo* (**Figure 2**; Rhein et al., 2024). This is well known for other dicarbonyls, but it seems that dimethylglyoxal has even more severe effects than, for example, methylglyoxal. In the context of vascular dementia, decreased levels of the tight-junction protein occludin were demonstrated after dimethylglyoxal exposure *in vitro* and *in vivo*, indicating at least a contributory effect of dimethylglyoxal toxicity to blood–brain barrier disruption in diabetes. Strikingly, mice showed signs of mild cognitive impairment after long-term oral dimethylglyoxal treatment similar to diabetic animals (Rhein et al., 2024).

Experiments showed that dimethylglyoxal production is enhanced under hypoxic conditions (0.1%–0.5% O_2_) (Rhein et al., 2024). This is of particular interest, considering the pathology of hyperglycemic stroke. In the acute phase of ischemic stroke, approximately 40% of patients present with hyperglycemia on admission, even in the absence of a pre-existing diabetes. The acute stress-induced hyperglycemia worsens brain damage and consequently the outcome of patients. Unfortunately, regulating blood glucose levels with insulin has not shown the desired therapeutic effect in a clinical study (Johnston et al., 2019). Therefore, it is conceivable that downstream glucose metabolites are responsible for the observed pathology of hyperglycemic stroke, making dicarbonyls highly likely candidates. Indeed, in a mouse model of hyperglycemic stroke, dimethylglyoxal levels were slightly elevated in the plasma and tremendously increased in the brain with an even more dramatic effect in the ischemic hemisphere of the brain (Rhein et al., 2024). The concept that dicarbonyls contribute to the detrimental effect of hyperglycemia in acute stroke is supported by the role of the RAGE receptor (**Figure 2**). Hyperglycemia did not aggravate ischemic brain damage in RAGE-deficient mice but only in wild-type mice (Khan et al., 2016).

Endogenous dimethylglyoxal originates from two pyruvate molecules, but the individual enzymes responsible for dimethylglyoxal formation have not been identified yet. In bacteria, dimethylglyoxal is produced via acetolactate synthase as part of branch-chain amino acid synthesis. However, in mammals, this pathway is lost and branch-chain amino acids have become essential nutrients. The gene encoding the enzyme bacterial acetolactate synthase-like (ILVBL) has been conserved, though. Studies showed that under certain conditions, like long-term or ischemic hyperglycemia, an *Ilvbl* knock-out lowered dimethylglyoxal levels in the brain (Rhein et al., 2024). Moreover, a glycated amino acid originating from the reaction of dimethylglyoxal with lysine, or lysine-residues in proteins, was identified named Nε-3-hydroxy-2-butanonelysine (HBL). Interestingly, HBL levels in the brain were reduced in acute hyperglycemic *Ilvbl* knock-out animals as well. Nevertheless, even with the complete knock-out of *Ilvbl*, mice still displayed significant levels of dimethylglyoxal in plasma, peripheral tissue, and the brain. Other, not yet identified enzymes and pathways must therefore be involved in endogenous dimethylglyoxal production. One contributing factor could be dimethylglyoxal originating from microbiota. It is likely that microorganisms populating the gut produce considerable amounts of dimethylglyoxal. However, due to its high reactivity, it is not expected that dimethylglyoxal produced in the gut would be detected in the brain. Even though dimethylglyoxal is able to cross the intestinal as well as the blood–brain barrier (Rhein et al., 2024), local production by cerebral cells is more likely. Unraveling the metabolic systems, next to ILVBL, that are responsible for dimethylglyoxal formation would not only advance the understanding of dimethylglyoxal production, but could also reveal further pharmacological targets to reduce dimethylglyoxal levels, especially in states of acute or chronic hyperglycemia.

Instead of targeting dimethylglyoxal directly, intervening with pyruvate availability is a promising approach. Metformin, a long known and established drug in diabetes, inhibits the mitochondrial glycerol-3-phosphate dehydrogenase, shifting the lactate to pyruvate ratio towards lactate (Madiraju et al., 2014). Consequently, reducing levels of pyruvate would prevent excessive dimethylglyoxal production. In fact, the combination of insulin and metformin was most effective in reducing dimethylglyoxal serum levels in patients with diabetes compared to insulin or metformin alone (Rhein et al., 2024). Even more promising might be the implementation of metformin for treating hyperglycemic stroke. With insulin being not effective, despite lowering blood glucose levels, metformin could help attenuate the detrimental consequences of acute hyperglycemia on the ischemic brain tissue by hindering dimethylglyoxal production. Intriguingly, preclinical data support this concept (Guo et al., 2023).

The discovery of the role of dimethylglyoxal in diabetes and hyperglycemic stroke raises new questions waiting to be answered. The role of dimethylglyoxal in peripheral neuropathy and nerve regeneration remains to be investigated. Published research indicates that reducing dicarbonyl levels, including dimethylglyoxal, mitigates vascular pathologies related to hyperglycemia (Singh et al., 2001; Rhein et al., 2024). Strategies such as dietary interventions, lifestyle changes, and pharmacological agents are being explored to manage dicarbonyl levels effectively (Singh et al., 2001; Rhein et al., 2024). Regarding the role of dimethylglyoxal, further studies will be necessary to specify the enzymes and pathways involved in production and maybe even elimination of dimethylglyoxal. This will open up new possibilities for the protection of specific cell types from dimethylglyoxal-mediated dicarbonyl stress. More comprehensive analyses of the effect of metformin on pyruvate and dimethylglyoxal levels are needed, including the analysis of larger clinical cohorts.

In most analyses, dicarbonyls are measured by LC-MS/MS after derivatization to reduce their polarity and ensure reliable detection. LC-MS/MS is a powerful tool that is also the state-of-the-art technique for the untargeted analysis of metabolites. Unfortunately, due to their low molecular mass and high polarity, dicarbonyls are difficult to detect by commonly used LC-MS/MS-based metabolomics screens as they are more prone to signal-to-noise interferences and inadequate retention. In the future, appropriate and more targeted approaches may lead to the discovery of additional unknown sugar metabolites and their glycation products, promoting diabetes research and therapeutic approaches. Proteomics approaches could be useful to detect glycated sites of proteins to assess the total extent of protein modification by dicarbonyls like dimethylglyoxal.

In summary, dicarbonyls are significant players in the pathology of diabetes. However, so far, there is no specific treatment targeting dicarbonyl stress beyond general lowering of blood glucose levels. The discovery of dimethylglyoxal in the context of glucose-mediated brain damage via pyruvate highlights the importance of understanding its role in disease progression and the potential for therapeutic interventions aimed at reducing dicarbonyl impact.

*This work has been supported by funding from the Deutsche Forschungsgemeinschaft (SCHW 416/7-1) and from the European Research Council (ERC) under the European Union’s Horizon 2020 research and innovation program (grant agreement No. 810331) (to MS).*
